# Secure Multiuser Communications in Wireless Sensor Networks with TAS and Cooperative Jamming

**DOI:** 10.3390/s16111908

**Published:** 2016-11-12

**Authors:** Maoqiang Yang, Bangning Zhang, Yuzhen Huang, Nan Yang, Daoxing Guo, Bin Gao

**Affiliations:** 1 College of Communications Engineering, PLA University of Science and Technology, No. 2 Biaoying, Qinhuai District, Nanjing 210007, China; yyypub@163.com (M.Y.); zbnpub@163.com (B.Z.); yzh_huang@sina.com (Y.H.); feimaxiao123@gmail.com (B.G.); 2 Research School of Engineering, Australian National University, Canberra, ACT 2601, Australia; nan.yang@anu.edu.au

**Keywords:** wireless sensor networks, physical layer security, multiuser scheduling, transmit antenna selection, cooperative jamming, secrecy outage probability, effective secrecy throughput

## Abstract

In this paper, we investigate the secure transmission in wireless sensor networks (WSNs) consisting of one multiple-antenna base station (BS), multiple single-antenna legitimate users, one single-antenna eavesdropper and one multiple-antenna cooperative jammer. In an effort to reduce the scheduling complexity and extend the battery lifetime of the sensor nodes, the switch-and-stay combining (SSC) scheduling scheme is exploited over the sensor nodes. Meanwhile, transmit antenna selection (TAS) is employed at the BS and cooperative jamming (CJ) is adopted at the jammer node, aiming at achieving a satisfactory secrecy performance. Moreover, depending on whether the jammer node has the global channel state information (CSI) of both the legitimate channel and the eavesdropper’s channel, it explores a zero-forcing beamforming (ZFB) scheme or a null-space artificial noise (NAN) scheme to confound the eavesdropper while avoiding the interference to the legitimate user. Building on this, we propose two novel hybrid secure transmission schemes, termed TAS-SSC-ZFB and TAS-SSC-NAN, for WSNs. We then derive the exact closed-form expressions for the secrecy outage probability and the effective secrecy throughput of both schemes to characterize the secrecy performance. Using these closed-form expressions, we further determine the optimal switching threshold and obtain the optimal power allocation factor between the BS and jammer node for both schemes to minimize the secrecy outage probability, while the optimal secrecy rate is decided to maximize the effective secrecy throughput for both schemes. Numerical results are provided to verify the theoretical analysis and illustrate the impact of key system parameters on the secrecy performance.

## 1. Introduction

Wireless sensor networks (WSNs) are envisioned as an emerging research field with numerous applications, such as health monitoring, vehicular tracking, military surveillance and environment sensing. Therefore, the research of WSNs has recently attracted a tremendous amount of attention from both industry and academia [1,2]. Generally, in the WSNs, a large number of the sensor nodes are deployed to collect the environmental information, and then report the sensed data to a base station (BS) wirelessly [3,4]. However, the secure transmission of WSNs is a fundamental concern due to the broadcast characteristics of radio propagation, and thus the sensing information is required to be safeguarded [5,6]. Conventionally, the cryptographic encryption relying on a secrecy key is broadly adopted to protect the confidential message from being wiretapped by the eavesdroppers. Nevertheless, the limitations behind the traditional cryptographic techniques lie in the complex protocols and architectures for the distribution and management of secret keys. It is noteworthy that the sensor nodes are the energy-constrained, cost-constrained, and lightweight computing devices, in which a considerable portion of the available energy is allocated to support the core sensorial and computational capabilities. Hence, there is possibly little left over to provide the security [7,8,9,10,11,12]. As such, it is of interest to explore efficient and low-complexity protocols to guarantee the secrecy of WSNs.

To address the above concerns, the physical layer security (PLS) technique has emerged as an attractive approach to achieve the perfect secrecy from an information-theoretical perspective [13]. The basic idea of PLS is to take advantage of the imperfection of wireless medium (e.g., fading, interference and noise) to ensure the secure transmission between the legitimate parties. By introducing randomness and structured redundancy into the data signal, the PLS enables legitimate users to decode the confidential messages correctly while keeps the eavesdropper from extracting the messages successfully [4,14,15].

Recently, various advanced techniques such as multi-antenna, cooperative relaying and cooperative jamming have been incorporated to further boost the potential benefits of PLS. In particular, transmit antenna selection (TAS) has been widely investigated on account of the low realization complexity of radio frequency (RF) chain, meanwhile yielding full diversity [16,17,18,19,20,21]. Recently, Yang et al. [16] proposed and analyzed TAS to enhance PLS in multiple-input multiple-output (MIMO) wiretap channels. Later on, TAS with Alamouti coding and power allocation was addressed in [17]. Considering the outdated channel state information (CSI) due to feedback delay, the authors in [18] investigated the secrecy outage performance of spectrum sharing MIMO networks with generalized TAS and maximal ratio combining (MRC) over the Nakagami-*m* channel. Based on whether the source node has the global CSIs of both the main link and eavesdropper’s link, optimal antenna selection (OAS) and suboptimal antenna selection (SAS) were proposed in [19] with the traditional space-time transmission (STT) as a benchmark in MIMO systems. Meanwhile, [20] examined TAS/MRC and TAS/selection combining (SC) scheme with decode-and-forward (DF) relaying in underlay spectrum sharing with multiple primary users (PU) transceivers and multiple antennas at the secondary users (SUs). In addition, in [21], the secrecy performance of multiple-input single-output (MISO) simultaneous wireless information and the power transfer (SWIPT) system was studied with TAS and imperfect CSI.

In parallel, cooperative jamming has been identified as an effective paradigm to enhance the security due to its ability of reducing the leakage rate to the wiretapper. Loosely speaking, the jamming signals can be transmitted from the source [14,15], the legitimate destination [22,23] and the relay [24,25,26,27,28,29,30]. As indicated in these studies, the jamming signals need to be designed carefully since the interference may also be leaked to the desired user. With the assistance of artificial noise, the optimal secure transmission was addressed by considering an on-off transmission scheme and an adaptive transmission scheme in the MISO single-antenna eavesdropper wiretap channel [14] and in the MISO multi-antenna eavesdropper wiretap channel [15], respectively. In [14,15], the artificial noise was transmitted in conjunction with the information signal at the BS, and beamforming matrix was designed to deteriorate the eavesdropper’s channel quality by transmitting noise in all directions except towards the intended user. Furthermore, [22] generated the artificial jamming noise at the legitimate receiver, under the assumption that the receiver knows the artificial jamming noise and thus can cancel it by performing self-interference subtraction. In [23], a joint scheme of destination-aid cooperative jamming and precoding at both the source and the relay was proposed for dual-hop amplify-and-forward MIMO untrusted relay systems, where the self-interference is assumed to be perfectly estimated and can be subtracted from the received signal. In addition, considering the jamming signals emitted by the relay, the external helper degraded the eavesdropper’s channel without hurting the legitimate channel. With imperfect CSI, the secure communication aided by a multi-antenna cooperative jammer was addressed in [24]. Taking into account which role the helper should take to enhance the secrecy, [25] investigated a direct transmission scheme (DTS) and a relay transmission scheme (RTS) in terms of ergodic secrecy rate and optimal power allocation. The work in [26] investigated different secrecy rate optimization techniques for a multi-antenna cooperative jammer assisted MIMO secrecy channel. Moreover, in [27], three secure transmission schemes were investigated in multi-antenna relay systems with cooperative jamming in terms of the ergodic achievable secrecy rate. Very recently, the worst-case cooperative jamming for secure communications in the cognitive internet of things (CIoT) was investigated in [28]. In [29], the MRC/ZFB scheme at the relay was designed to enhance the secrecy performance of dual-hop multi-antenna spectrum sharing relaying networks, while the cooperative jamming with the ZFB scheme was addressed in [30] to achieve secure transmission in cooperative relaying networks.

It is critical to note that, in multiuser communication systems, the conventional opportunistic scheduling scheme requires the feedback of channel information for all the diversity branches. Based on the continuously-updated CSIs of all the nodes in the network, full multiuser diversity gains are explored at the central scheduler. However, a significant portion of the battery energy of the low-end terminals and a large share of air-link resources are occupied to feed the CSIs back instead of valuable data traffic [31]. To circumvent this difficulty, the multiuser switched diversity scheduling schemes were proposed in [32] in order to search any acceptable user (i.e., with good channel quality) rather than the best one among all. Recently, PLS with threshold-based multiuser scheduling was studied in multi-antenna wireless networks [33]. Considering the imperfect decoding at the regenerative relay, the secure multiuser scheduling was investigated in dual-hop relay networks over Nakagami-*m* fading in [34]. In particular, the multi-branch switch-and-stay combining (SSC) scheme was first addressed in [35], which reduces the implementation complexity for multi-channel communication scenarios. In the multi-branch SSC scheme, if the channel quality of the currently connected branch exceeds a predetermined threshold, then this branch is kept. Otherwise, no matter what the channel quality of the switch-to branch is, the scheduler settles on that branch for the next transmission burst [35]. It is noteworthy that, in considering the opportunistic relay selection in cooperative networks, the distributed SSC scheme was explored in [36] for secrecy enhancement. More recently, a secure SSC protocol was proposed in [37] to overcome the high relay switching rate for two-phase underlay cognitive relay networks.

To the best knowledge of the authors, the SSC based secure multiuser transmission with TAS and cooperative jamming for WSNs has not been reported in literature thus far. We are therefore motivated to examine the security level of such networks when cooperative jamming is applied in parallel with user selection. The main contributions of this paper are summarized as follows:

- Two novel hybrid secure transmission schemes, i.e., TAS-SSC-ZFB and TAS-SSC-NAN, are proposed for securing the data transmission in WSNs while achieving low feedback requirements and examination costs.
- Exact closed-form expressions for the secrecy outage probability and effective secrecy throughput are derived for the proposed schemes, which provide an efficient and convenient approach to characterize the secrecy performance of the considered network.
- Using these closed-form expressions, the optimal switching threshold is determined and the optimal power allocation factor between the BS and CJ is obtained for both schemes to minimize the secrecy outage probability. In addition, the optimal secrecy rate is decided for both schemes to maximize the effective secrecy throughput. Our findings demonstrate that the TAS-SSC-ZFB scheme outperforms the TAS-SSC-NAN scheme in terms of both secrecy outage probability and effective secrecy throughput, while the TAS-SSC-NAN scheme is more robust than the TAS-SSC-ZFB scheme.

The remaining parts of the paper are organized as follows. In Section 2, the system model and transmission protocols of TAS-SSC-ZFB and TAS-SSC-NAN are presented, and the secrecy performance of both schemes are analyzed in Section 3. In Section 4, the numerical simulation and discussions are provided to validate the theoretical analysis. Finally, the conclusions are drawn in Section 5.

## 2. System Model and Transmission Protocol

### 2.1. System Model

**Figure 1:**
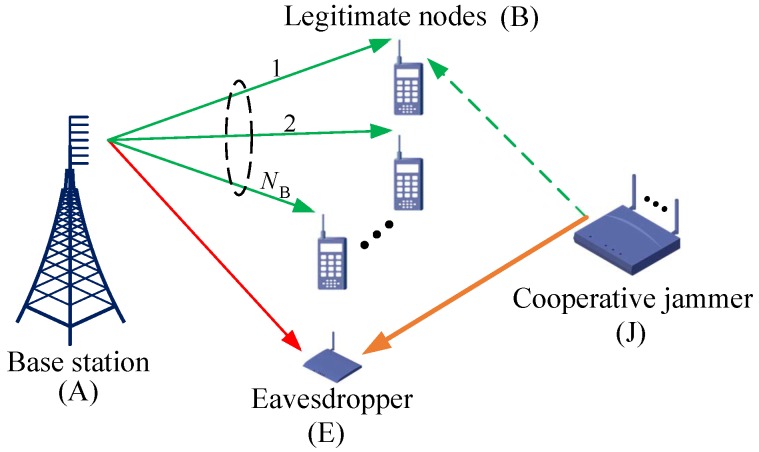
System model.

Let us consider a multiuser downlink wireless sensor network, as illustrated in Figure 1, in which a base station (A) with $A_{A}$ transmit antennas serves $N_{B}$ single-antenna legitimate sensor nodes (B) in the presence of a single-antenna eavesdropper (E), and a friendly pure jammer (J) with $A_{J}$ antennas $\left(A_{J}\geq 2\right)$. In this model, we preserve the practical assumption that the legitimate channel, jammer’s channel and the eavesdropper’s channel are subject to independent and non-identically distributed (i.n.i.d) flat Rayleigh fading such that they have different average signal-to-noise ratio (SNR), i.e., ${\bar{\gamma }}_{B},{\bar{\gamma }}_{J}$ and ${\bar{\gamma }}_{E}$, and the involved fading coefficients are quasi-stationary within the channel coherence time. In order to perform secure transmission, the BS encodes the messages with a capacity achieving wiretap codebook and then transmits the resulting codewords to the legitimate user. In addition, each transmission block is considered to be equivalent to the channel coherence time and is composed of two parts, i.e., guard time and data transmission time.

### 2.2. Secure Transmission Schemes

We now detail the proposed secure transmission schemes in the considered WSNs. In general, the complete transmission procedure can be separated into two phases.

In the first phase, an acceptable user of $N_{B}$ candidates is selected according to the SSC scheme [35] out of $A_{A}$ transmit antennas within the guard time to carry out the data transmission. To be specific, considering the first transmit antenna (α=1), the previously selected user *k* $\left(k\in \left\{1,2,\ldots ,N_{B}\right\}\right)$ compares its instantaneous SNR $\gamma_{\alpha ,k}^{b}$ with the pre-determined switching threshold $\gamma_{T}$. If the received instantaneous SNR exceeds $\gamma_{T}$, then it stays without switching over and feeds the SNR and user index back to the BS with $\gamma_{B,\alpha }=\gamma_{\alpha ,k}^{b}\geq \gamma_{T}$. Otherwise, it switches to the next user regardless of its SNR following the similar feedback operation with $\gamma_{B,\alpha }=\gamma_{\alpha ,k+1}^{b}$. The same SSC operation repeats for the rest of $\left.A_{A}-1\right.$ transmit antennas.

To proceed, the pair of transmit antenna and corresponding selected user that gives the largest instantaneous SNR is picked out, which is right for the data transmission time. As such, only one legitimate user is scheduled for data transmission without continuously examining all the users in the WSNs, which brings about considerable savings of feedback requirements and implementation complexity. Therefore, the selected antenna is given by

(1) $$\begin{array}{c} \alpha_{}^{*}=\underset{1\leq \alpha \leq A_{A}}{\mathrm{argmax}}\left(\gamma_{B,\alpha }^{}\right). \end{array}$$

In the second phase, we consider a cooperative relay node, which serves as the friendly pure jammer. Note that the synchronization requirement between the cooperative jammer and the BS can be implemented by some well-known techniques, for instance, the time-service from the GPS or compass. Alternatively, the BS can broadcast the timing information, which enables the cooperative jammer to keep the same pace with the BS. Depending on whether the relay node has the global CSIs of both J → B link and J → E link, the ZFB scheme and NAN scheme are, respectively, explored to confound the eavesdropper while avoiding interference with the selected legitimate user.

#### 2.2.1. Zero-Forcing Beamforming

Firstly, similar to [29,30,38,39], we assume that the CSIs of both J → B link and J → E link are available at the relay. It is pointed out that this scenario is reasonable in the multiuser system where the user may play dual roles as legal receiver for some messages and as eavesdropper for others [40,41,42].

The purpose of the ZFB scheme is to maximize the interference imposed on the eavesdropper while avoiding the interruption to the selected legitimate user. To this end, according to the principle of ZFB scheme, we obtain

(2) $$\begin{array}{c} \begin{array}{c} \max_{w}\left|h_{\mathrm{JE}}^{†}w\right|,\\ s.t.\left|h_{\mathrm{JB}}^{†}w\right|=0\;\&\;{∥w∥}_{F}=1, \end{array} \end{array}$$

where † denotes the conjugate transpose operator and ${∥\cdot ∥}_{F}$ represents the Frobenius norm. w is the weight vector, $h_{\mathrm{JB}}$ and $h_{\mathrm{JE}}$, separately, denote the $A_{J}\times 1$ vector for the CSIs of J → B link and J → E link, whose entries follow Rayleigh distribution with zero mean, and variance $\lambda_{\mathrm{JB}}$ and $\lambda_{\mathrm{JE}}$, respectively.

Based on the projection matrix theory [43] (Proposition 1), the optimum beamforming vector w is given by

(3) $$\begin{array}{c} w=\frac{{ℵ}^{⊥}h_{\mathrm{JE}}^{}}{{∥{ℵ}^{⊥}h_{\mathrm{JE}}^{}∥}_{F}^{}}, \end{array}$$

where ${ℵ}^{⊥}=\left.I-h_{\mathrm{JB}}{\left(h_{\mathrm{JB}}^{†}h_{\mathrm{JB}}\right)}^{-1}h_{\mathrm{JB}}^{†}\right.$ is the projection idempotent matrix. Hence, the instantaneous SNR of the legitimate channel is given by

(4) $$\begin{array}{c} \gamma_{B}^{}=\frac{P_{A}}{\sigma_{B}^{2}}{\left|h_{\alpha^{*}B}\right|}^{2}, \end{array}$$

and the instantaneous received signal-to-interference-and-noise ratio (SINR) of eavesdropper’s channel is given by

(5) $$\begin{array}{c} \gamma_{E}^{\left(†\right)}=\frac{\frac{P_{A}^{}}{\sigma_{E}^{2}}{\left|h_{\alpha^{*}E}\right|}^{2}}{\frac{P_{J}^{}}{\sigma_{E}^{2}}{∥{ℵ}^{⊥}h_{\mathrm{JE}}^{}∥}_{F}^{2}+1}, \end{array}$$

where $h_{\alpha^{*}B}$ and $h_{\alpha^{*}E}$ denote the CSIs of the selected legitimate channel and the selected transmit antenna to the eavesdropper channel, respectively. Its entries follow Rayleigh distribution with zero mean, and variance $\lambda_{\mathrm{AB}}$ and $\lambda_{\mathrm{AE}}$. $\sigma_{B}^{2}$ and $\sigma_{E}^{2}$ denote the noise variance at the legitimate user and eavesdropper, respectively. $P_{A}$ and $P_{J}$ denote the transmit powers at the BS and the jammer, respectively.

#### 2.2.2. Null-Space Artificial Noise

To relax the assumption in the ZFB scheme, we now consider that the CSI of J → E link is unavailable at the relay and the relay only has the knowledge of CSI for J → B link. Here, the NAN scheme is exploited to emit artificial noise in the nullspace of the selected legitimate user but disperse in all directions towards the eavesdropper. We design the $A_{J}\times A_{J}$ beamforming matrix as $W=\left[w_{\mathrm{JB}},W_{\mathrm{JE}}\right]$, where $w_{\mathrm{JB}}$ is an $A_{J}\times 1$ vector used for J → B link and $W_{\mathrm{JE}}$ is an $A_{J}\times \left(A_{J}-1\right)$ matrix used to deteriorate the quality of eavesdropper’s channel by transmitting AN in all directions except towards the selected legitimate user.

To do so, we choose $w_{\mathrm{JB}}$ as the principle eigenvector corresponding to the largest eigenvalue of $h_{\mathrm{JB}}h_{\mathrm{JB}}^{†}$, and then choose $W_{\mathrm{JE}}$ as the remaining $A_{J}-1$ eigenvectors of $h_{\mathrm{JB}}h_{\mathrm{JB}}^{†}$ such that $W_{\mathrm{JE}}$ lies in the nullspace of $h_{\mathrm{JB}}$, i.e., $h_{\mathrm{JB}}W_{\mathrm{JE}}=0$. As such, W is a unitary matrix. In addition, since the cooperative jammer has no knowledge about $h_{\mathrm{JE}}$, the jammer distributes the transmit power $P_{J}$ uniformly across the $A_{J}-1$ transmit antennas. Building on this, we have the same instantaneous SNR of the legitimate channel as the ZFB scheme, and the corresponding instantaneous SINR of the eavesdropper’s channel is given by

(6) $$\begin{array}{c} \gamma_{E}^{\left(‡\right)}=\frac{\frac{P_{A}^{}}{\sigma_{E}^{2}}{\left|h_{\alpha^{*}E}\right|}^{2}}{\frac{1}{A_{J}-1}\frac{P_{J}}{\sigma_{E}^{2}}{∥h_{\mathrm{JE}}W_{\mathrm{JE}}∥}_{F}^{2}+1}. \end{array}$$

For both cooperative jamming schemes, we further assume that the total transmit power adopted at the BS and CJ is constrained by $P_{S}$, i.e., $P_{A}+P_{J}=P_{S}$. We define ϕ, 0<ϕ<1, as the power allocation scaling factor which denotes the fraction of the power allocation to the BS, such that $P_{A}=\phi P_{S}$ and $P_{J}=\left(1-\phi \right)P_{S}$. For notational convenience, we define the overall average transmit SNR as ${\bar{\gamma }}_{S}=P_{S}/\sigma_{B}^{2}$, the average transmit SNR of A→B link, A→E link and J→E link as ${\bar{\gamma }}_{B}=P_{A}/\sigma_{B}^{2}$, ${\bar{\gamma }}_{E}=P_{A}/\sigma_{E}^{2}$ and ${\bar{\gamma }}_{J}=P_{J}/\sigma_{E}^{2}$, respectively.

We also denote $M_{\mathrm{BE}}={\bar{\gamma }}_{B}/{\bar{\gamma }}_{E}$ as the ratio between the average SNR of A→B link and A→E link, i.e., the main-to-eavesdropper (MER) ratio. As such, we have ${\bar{\gamma }}_{B}=\phi {\bar{\gamma }}_{S}$, ${\bar{\gamma }}_{E}={\bar{\gamma }}_{B}/M_{\mathrm{BE}}$ and ${\bar{\gamma }}_{J}=\left(1-\phi \right){\bar{\gamma }}_{S}/M_{\mathrm{BE}}$.

We highlight that the proposed methods bring about several advantages that are of particular interest to WSNs. First, the exploitation of SSC scheduling among legitimate nodes help to avoid a large amount of CSI feedback, which, in turn, reduces the share of air-link resources. Second, the best antenna at the BS is optimal to the selected legitimate link but is equivalent to a random transmit antenna for the eavesdropper. Thus, the eavesdropper cannot achieve any transmit diversity from the best antenna. Third, the cooperative jamming schemes, i.e., the ZFB scheme and NAN scheme, are designed to increase the interference at the eavesdropper while avoiding interrupting the selected legitimate node, which provides a further safeguard for data transmission in WSNs. Building on these advantages, we clarify that our proposed schemes allow low-cost sensor nodes to fully explore the limited battery energy to support the core sensorial and computational operations, while guaranteeing the secure data transmission from the sensor nodes.

### 2.3. Achievable Secrecy Rate

Now, the achievable secrecy rate of the SSC based WSNs with TAS and cooperative jamming is provided by

(7) $$\begin{array}{c} \begin{array}{c} C_{s}={\left[C_{B}^{}-C_{E}\right]}^{+}=\left.[\log\left(1+\gamma_{B}\right)-\log\left(1+\gamma_{E}\right)\right.{]}^{+} \end{array}, \end{array}$$

where ${\left[u\right]}^{+}=\max\left(u,0\right)$, $C_{B}=\log\left(1+\gamma_{B}\right)$ and $C_{E}=\log\left(1+\gamma_{E}\right)$ represent the instantaneous capacity of the legitimate channel and eavesdropper’s channel, respectively.

## 3. Secrecy Performance Analysis

In this section, we analyze the secrecy outage probability and the effective secrecy throughput as the main performance metrics to examine the secrecy performance of the considered network.

### 3.1. Preliminaries

Before proceeding, we first determine the statistic properties of the end-to-end instantaneous SNRs in the considered network. According to the basic principle of the SSC scheme and following the same steps developed in [35], the cumulative distribution function (CDF) for $\gamma_{B,\alpha }$ is provided by

(8) $$\begin{array}{c} F_{\gamma_{B,\alpha }^{}}\left(\gamma \right)=\left\{\begin{array}{cc} F_{\gamma_{\alpha ,k}^{b}}\left(\gamma_{T}\right)F_{\gamma_{\alpha ,k}^{b}}\left(\gamma \right), & \gamma <\gamma_{T},\\ F_{\gamma_{\alpha ,k}^{b}}\left(\gamma \right)+F_{\gamma_{\alpha ,k}^{b}}\left(\gamma_{T}\right)\left[F_{\gamma_{\alpha ,k}^{b}}\left(\gamma \right)-1\right], & \gamma \geq \gamma_{T}. \end{array}\right. \end{array}$$

Moreover, according to the antenna selection at the BS, we define $\gamma_{B}$ as the resulting instantaneous SNR of A → B link with $\gamma_{B}=\max\left\{\gamma_{B,\alpha }\right\},\alpha \in \left\{1,2,\ldots ,A_{A}\right\}$. Considering that each legitimate channel is subject to independent and identical distribution (i.i.d.) Rayleigh fading, the CDF of $\gamma_{B}$ is given by $F_{\gamma_{B}}\left(\gamma \right)={\left[F_{\gamma_{B,\alpha }}\left(\gamma \right)\right]}^{A_{A}}$. Furthermore, with the assistance of binomial theorem [44] (Equation (1.111)), the CDF of $\gamma_{B}$ can be re-expressed as

(9) FγBγ=Fγα,kbγTAAFγα,kbγAA,γ<γT,∑q=0AAAAqFγα,kbγTq∑q1=0qqq1−1q1Fγα,kbγAA−q1,γ≥γT,

where $F_{\gamma_{\alpha ,k}^{b}}\left(\gamma \right)=1-\frac{\gamma }{{\bar{\gamma }}_{B}}\exp\left(-\frac{\gamma }{{\bar{\gamma }}_{B}}\right)$ denotes the CDF for end-to-end instantaneous SNR of each legitimate link.

On the other hand, according to Equation (5), the probability density function (PDF) of end-to-end instantaneous SNR $\gamma_{E}$ with ZFB scheme can be expressed as

(10) $$\begin{array}{c} f_{\gamma_{E}}^{\left(†\right)}\left(x\right)=\frac{1}{{\bar{\gamma }}_{E}}{\left(1+\frac{{\bar{\gamma }}_{J}}{{\bar{\gamma }}_{E}}x\right)}_{}^{-\left(A_{J}-1\right)}\exp\left(-\frac{x}{{\bar{\gamma }}_{E}}\right)\left[\left(A_{J}-1\right){\bar{\gamma }}_{J}{\left(1+\frac{{\bar{\gamma }}_{J}}{{\bar{\gamma }}_{E}}x\right)}_{}^{-1}+1\right]. \end{array}$$

Similarly, based on Equation (6), the PDF of end-to-end instantaneous SNR $\gamma_{E}^{\left.\right.}$ with NAN scheme is given by

(11) $$\begin{array}{c} f_{\gamma_{E}}^{\left(‡\right)}\left(x\right)=\frac{1}{{\bar{\gamma }}_{E}}{\left(1+\frac{{\bar{\gamma }}_{J}}{{\bar{\gamma }}_{E}}\frac{1}{A_{J}-1}x\right)}_{}^{-\left(A_{J}-1\right)}\exp\left(-\frac{x}{{\bar{\gamma }}_{E}}\right)\left[{\bar{\gamma }}_{J}{\left(1+\frac{{\bar{\gamma }}_{J}}{{\bar{\gamma }}_{E}}\frac{1}{A_{J}-1}x\right)}_{}^{-1}+1\right]. \end{array}$$

**Proof.**  The detailed derivation of Equation (10) can be found in [30] (Appendix D), and following the similar lines we have Equation (11). ☐

### 3.2. Secrecy Outage Probability

The definition of secrecy outage probability is the probability that the achievable secrecy rate falls down the predetermined secrecy rate $R_{s}$ [30,38,41]. Mathematically, the secrecy outage probability can be formulated as

(12) $$\begin{array}{c} O_{\mathrm{out}}\left(R_{s}\right)=\mathrm{Pr}\left(C_{s}<R_{s}\right). \end{array}$$

Now, an exact closed-form expression for secrecy outage probability of the considered system is derived and expressed in the following theorem.

**Theorem** **1.** *The secrecy outage probability of the SSC based WSNs with TAS and cooperative jamming is derived as*

(13) $$\begin{array}{c} O_{\mathrm{out}}^{\left(\kappa \right)}\left(R_{s}\right)=\left\{\begin{array}{cc} O_{\mathrm{out}-A}^{\left(\kappa \right)}\left(R_{s}\right), & \Pi_{\gamma_{T}}\geq 0,\\ O_{\mathrm{out}-B}^{\left(\kappa \right)}\left(R_{s}\right), & \Pi_{\gamma_{T}}<0, \end{array}\right. \end{array}$$

*where $\kappa \in \left\{I,\mathrm{II}\right\}$ with I and II stand for the TAS-SSC-ZFB scheme and the TAS-SSC-NAN scheme, respectively. $O_{\mathrm{out}}^{\left(I\right)}\left(R_{s}\right)$ and $O_{\mathrm{out}}^{\left(\mathrm{II}\right)}\left(R_{s}\right)$ are provided by Equation (19) and Equation (20), as shown at the top of the next page, respectively. $z=\frac{2^{R_{s}}q_{2}}{{\bar{\gamma }}_{B}}+\frac{1}{{\bar{\gamma }}_{E}}$, $E_{1}=\exp\left(-\frac{2^{R_{s}}-1}{{\bar{\gamma }}_{B}}\right)$, and $\Pi_{\gamma_{T}}=2^{-R_{s}}\left(1+\gamma_{T}\right)-1$. $\Gamma \left(\alpha ,\beta \right)$ and $\Psi \left(\mu ,\upsilon ;\tau \right)$ denote the upper incomplete Gamma function [44] (Equation (8.350.2)) and confluent hypergeometric function of the second kind [44] (Equation (9.211.4)), respectively.*

**Proof of Theorem** **1.** Based on the definition in Equation (12), we have

(14) $$\begin{array}{c} O_{\mathrm{out}}\left(R_{s}\right)=\underset{P_{1}}{\underset{︸}{\mathrm{Pr}\left(C_{s}<R_{s}|\gamma_{B}>\gamma_{E}\right)\mathrm{Pr}\left(\gamma_{B}>\gamma_{E}\right)}}+\underset{P_{2}}{\underset{︸}{\mathrm{Pr}\left(\gamma_{B}<\gamma_{E}\right)}}, \end{array}$$

where $P_{1}$ and $P_{2}$ are given by, respectively,

(15) $$\begin{array}{c} P_{1}=\int_{0}^{\infty }\int_{y}^{2^{R_{s}}\left(1+y\right)-1}f_{\gamma_{B}}\left(x\right)f_{\gamma_{E}}\left(y\right)dxdy \end{array}$$

and

(16) $$\begin{array}{c} P_{2}=\int_{0}^{\infty }\int_{0}^{y}f_{\gamma_{B}}\left(x\right)f_{\gamma_{E}}\left(y\right)dxdy. \end{array}$$

Now, inserting Equations (15) and (16) into Equation (14) for both schemes yields

(17) $$\begin{array}{c} \begin{array}{cc} O_{\mathrm{out}}^{}\left(R_{s}\right) & =\int_{0}^{\infty }\int_{0}^{2^{R_{s}}\left(1+y\right)-1}f_{\gamma_{B}}\left(x\right)f_{\gamma_{E}}^{}\left(y\right)dxdy=\int_{0}^{\infty }F_{\gamma_{B}}\left(2^{R_{s}}\left(1+y\right)-1\right)f_{\gamma_{E}}^{}\left(y\right)dy, \end{array} \end{array}$$

where $f_{\gamma_{B}}\left(x\right)$ is the PDF of $\gamma_{B}$. Owing to the fact that the switching threshold $\gamma_{T}$ is exploited in the CDF of $\gamma_{B}$ in Equation (8), we have the relationship between $2^{R_{s}}\left(1+y\right)-1$ and $\gamma_{T}$ in Equation (17), i.e., $2^{R_{s}}\left(1+y\right)-1\geq \gamma_{T}$ or $2^{R_{s}}\left(1+y\right)-1<\gamma_{T}$. For the simplicity of notation, we denote a boundary point by $\Pi_{\gamma_{T}}=2^{-R_{s}}\left(1+\gamma_{T}\right)-1$. To do so, the derivation of secrecy outage probability is separated into two parts regarding the bound point $\Pi_{\gamma_{T}}$. Thus, we have,

(18) $$\begin{array}{c} O_{\mathrm{out}}^{}\left(R_{s}\right)=\left\{\;\;\;\begin{array}{cc} \int_{0}^{\Pi_{\gamma_{T}}}F_{{}_{\gamma_{B}}}\left(\lambda_{y}^{}\right)f_{\gamma_{E}}^{}\left(y\right)dy+\int_{\Pi_{\gamma_{T}}}^{\infty }F_{{}_{\gamma_{B}}}\left(\lambda_{y}^{}\right)f_{\gamma_{E}}^{}\left(y\right)dy, & \Pi_{\gamma_{T}}\geq 0,\\ \int_{0}^{\infty }F_{{}_{\gamma_{B}}}\left(\lambda_{y}^{}\right)f_{\gamma_{E}}^{}\left(y\right)dy, & \Pi_{\gamma_{T}}<0, \end{array}\right. \end{array}$$

where $\lambda_{y}=2^{R_{s}}\left(1+y\right)-1$.

In the following, by substituting Equations (9) and (10) into Equation (18) and using [44] (Equations (1.111), (3.381.3), and (9.211.4)), the secrecy outage probability $O_{\mathrm{out}}\left(R_{s}\right)$ in Equation (18) is derived in Equation (19) for the TAS-SSC-ZFB scheme. Similarly, for the TAS-SSC-NAN scheme, $O_{\mathrm{out}}\left(R_{s}\right)$ is presented in Equation (20). ☐

We remark that our new derived expressions in Equations (19) and (20) are ready to compute because the involved functions are merely the easy-to-calculate exponential functions, power functions, upper incomplete Gamma functions and confluent hypergeometric functions. As such, the optimal performance and optimal parameters of the considered network are achieved with convenience.

We further highlight that the derived theoretical results in Equations (19) and (20) are valid for general WSNs with an arbitrary number of antennas at the BS and cooperative jammer, arbitrary number of legitimate users, arbitrary average SNRs and switching threshold.

(19a) Oout−AIRs=FγγTAA∑q=0AAAAq−1qE1qexpγ¯Ezγ¯Jγ¯Jγ¯Ezγ¯JAJ−1{AJ−1γ¯J[Γ1−AJ,γ¯Ezγ¯J−Γ1−AJ,γ¯Ezγ¯J+zΠγT]+γ¯Ezγ¯J−1Γ2−AJ,γ¯Ezγ¯J−Γ2−AJ,γ¯Ezγ¯J+zΠγT}+∑q=0AAAAqFγγTq∑q1=0qqq1−1q1∑q2=0AA−q1AA−q1q2−1q2E1q2expγ¯Ezγ¯Jγ¯Jγ¯Ezγ¯JAJ−1×AJ−1γ¯JΓ1−AJ,γ¯Ezγ¯J+zΠγT+γ¯Ezγ¯J−1Γ2−AJ,γ¯Ezγ¯J+zΠγT,

(19b) Oout−BIRs=∑q=0AAAAqFγγTq∑q1=0qqq1−1q1∑q2=0AA−q1AA−q1q2−1q2E1q2×AJ−1Ψ1,2−AJ;γ¯Eγ¯Jz+γ¯J−1Ψ1,3−AJ;γ¯Eγ¯Jz,

(20a) Oout−AIIRs=FγγTAA∑q=0AAAAq−1qE1qAJ−1γ¯JexpAJ−1γ¯Ezγ¯JAJ−1γ¯Ezγ¯JAJ−1×{γ¯JΓ1−AJ,AJ−1γ¯Ezγ¯J−Γ1−AJ,AJ−1γ¯Ezγ¯J+zΠγT+AJ−1γ¯Ezγ¯J−1×Γ2−AJ,AJ−1γ¯Ezγ¯J−Γ2−AJ,AJ−1γ¯Ezγ¯J+zΠγT}+∑q=0AAAAqFγγTq×∑q1=0qqq1−1q1∑q2=0AA−q1AA−q1q2−1q2E1q2AJ−1γ¯JexpAJ−1γ¯Ezγ¯JAJ−1γ¯Ezγ¯JAJ−1×γ¯JΓ1−AJ,AJ−1γ¯Ezγ¯J+zΠγT+AJ−1γ¯Ezγ¯J−1Γ2−AJ,AJ−1γ¯Ezγ¯J+zΠγT,

(20b) Oout−BIIRs=∑q=0AAAAqFγγTq∑q1=0qqq1−1q1∑q2=0AA−q1AA−q1q2−1q2E1q2×AJ−1Ψ1,2−AJ;γ¯EzAJ−1γ¯J+γ¯J−1Ψ1,3−AJ;γ¯EzAJ−1γ¯J.

### 3.3. Effective Secrecy Throughput

Before proceeding, we first present the definition of the secrecy transmission probability as the probability that the messages are confidentially conveyed from the BS to the legitimate user without leaking to the eavesdropper. Thus, according to [15] (Equation (8)), we have,

(21) $$\begin{array}{c} \begin{array}{c} P_{\sec}^{\left(\kappa \right)}\left(R_{s}\right)=1-O_{\mathrm{out}}^{\left(\kappa \right)}\left(R_{s}\right). \end{array} \end{array}$$

From Equation (21), it is found that the effective secrecy throughput can be characterized as the product of the secure transmission probability and secrecy rate $R_{s}$, which evaluates the average rate of the messages that are transmitted from the BS to the legitimate user confidentially in the passive wiretapping scenario [14,15]. We now present the effective secrecy throughput in the following theorem.

**Theorem** **2.** *The effective secrecy throughput of the SSC based WSNs with TAS and cooperative jamming is derived as*

(22) $$\begin{array}{c} S_{T}^{\left(\kappa \right)}\left(R_{s}\right)=\left\{\begin{array}{cc} \left(1-O_{\mathrm{out}-A}^{\left(\kappa \right)}\left(R_{s}\right)\right)R_{s}, & \Pi_{\gamma_{T}}\geq 0,\\ \left(1-O_{\mathrm{out}-B}^{\left(\kappa \right)}\left(R_{s}\right)\right)R_{s}, & \Pi_{\gamma_{T}}<0, \end{array}\right. \end{array}$$

*where $\kappa \in \left\{I,\mathrm{II}\right\}$ with I and II represent the TAS-SSC-ZFB scheme and the TAS-SSC-NAN scheme, respectively.*

**Proof of** **Theorem 2.** By substituting Equations (19) and (20) into Equation (22), the exact closed-form expressions for the effective secrecy throughput of the considered WSNs can be obtained. ☐

## 4. Simulations and Discussions

In this section, we perform the simulations with MATLAB R2014a 64-bit version (The MathWorks, Inc., Natick, MA, USA) running on a Windows 7 64-bit system (Microsoft, Redmond, Washington D.C., USA). Monte Carlo simulation results of the proposed TAS-SSC-ZFB and TAS-SSC-NAN schemes are presented to validate the conducted analysis and illustrate the joint impact of the key system parameters on the secrecy performance of the considered network.

**Figure 2:**
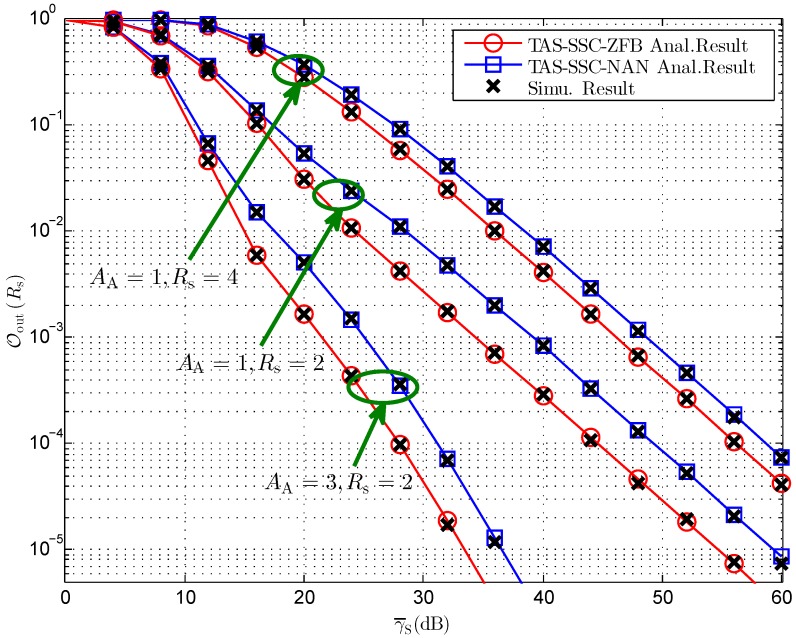
Secrecy outage probability versus different ${\bar{\gamma }}_{S}$ for $A_{J}=3$, $N_{B}=2,\gamma_{T}=10$ dB, $M_{\mathrm{BE}}=5$, and ϕ=0.5.

Figure 2 illustrates the secrecy outage probability versus overall average transmit SNR ${\bar{\gamma }}_{S}$ of the considered system with equal power allocation for both schemes. It can be readily observed that the theoretical analyses in Theorem 1 are in exact agreement with the Monte Carlo simulations, which demonstrates the correctness of our derived results. Moreover, we observe that the TAS-SSC-ZFB scheme outperforms the TAS-SSC-NAN scheme, which can be explained by the fact that the TAS-SSC-ZFB scheme benefits from the CSIs of both J → B channel and J → E channel while the TAS-SSC-NAN scheme merely relies on the knowledge of the J → B channel. In addition, as expected, increasing the number of transmit antennas brings about a significant secrecy performance improvement while enhancing the secrecy rate results in larger secrecy outage probability.

**Figure 3:**
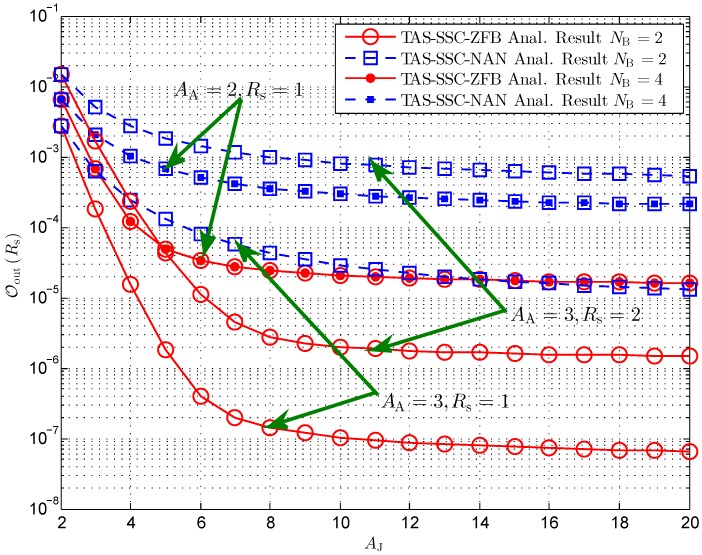
Secrecy outage probability versus different $A_{J}$ for $\gamma_{T}=10$ dB, $M_{\mathrm{BE}}=5$, ${\bar{\gamma }}_{S}=20$ dBW, and ϕ=0.5.

Figure 3 examines the secrecy outage probability versus different antenna configurations at the cooperative jammer. Interestingly, we first see that the TAS-SSC-ZFB scheme degrades into the TAS-SSC-NAN scheme when the number of antennas at the jammer is equal to two. Such an observation is explained by the fact that the PDF of $\gamma_{E}$ for the TAS-SSC-ZFB scheme is equivalent to that for the TAS-SSC-NAN scheme when $A_{J}=2$. Next, we find that the secrecy performance comes to a floor for both schemes when the number of antennas grows large. Notably, the secrecy outage floors can be further improved by increasing the number of antennas at the BS or decreasing the predetermined secrecy rate. Finally, we find that the secrecy performance is independent of the number of legitimate users in the networks. This is demonstrated by the observation that $O_{\mathrm{out}}\left(R_{s}\right)$ stays the same when $N_{B}=2$ increases to $N_{B}=4$ with $A_{A}=2,R_{s}=1$ setup for both schemes.

**Figure 4:**
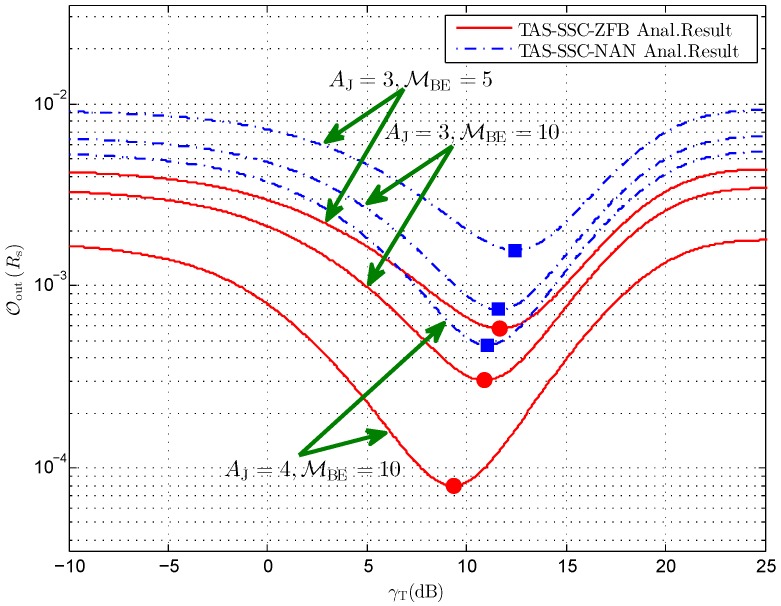
Secrecy outage probability versus different $\gamma_{T}$ for $A_{A}=2$, $N_{B}=2,R_{s}=1$, ${\bar{\gamma }}_{S}=20$ dB, and ϕ=0.5.

Figure 4 depicts the secrecy outage probability versus different switching threshold $\gamma_{T}$ for the given equal power allocation. Firstly, we find that there exists an optimal switching threshold $\gamma_{T}^{*}$ for both schemes, which accounts for the SSC scheduling being employed in the networks and being independent of the way that the cooperative jammer operates. Regarding the antenna configuration of the cooperative jammer, it can be observed that increasing the number of antennas has a positive impact on the secrecy performance and the minimum $O_{\mathrm{out}}\left(R_{s}\right)$ shifts to the left. In addition, we see that the secrecy outage probability degrades when ${\bar{\gamma }}_{E}$ increases and the optimal $\gamma_{T}^{*}$ shifts to right. For instance, $\gamma_{T}^{*}$ increases from 10.9 dB for $M_{\mathrm{BE}}=10$ to 11.7 dB for $M_{\mathrm{BE}}=5$ concerning the TAS-SSC-ZFB scheme when $A_{J}=3$, and increases from 11.6 dB for $M_{\mathrm{BE}}=10$ to 12.45 dB for $M_{\mathrm{BE}}=5$ concerning the TAS-SSC-NAN scheme when $A_{J}=3$. This reveals that a larger switching threshold setup is required to obtain the minimum $O_{\mathrm{out}}\left(R_{s}\right)$ for larger ${\bar{\gamma }}_{E}$.

**Figure 5:**
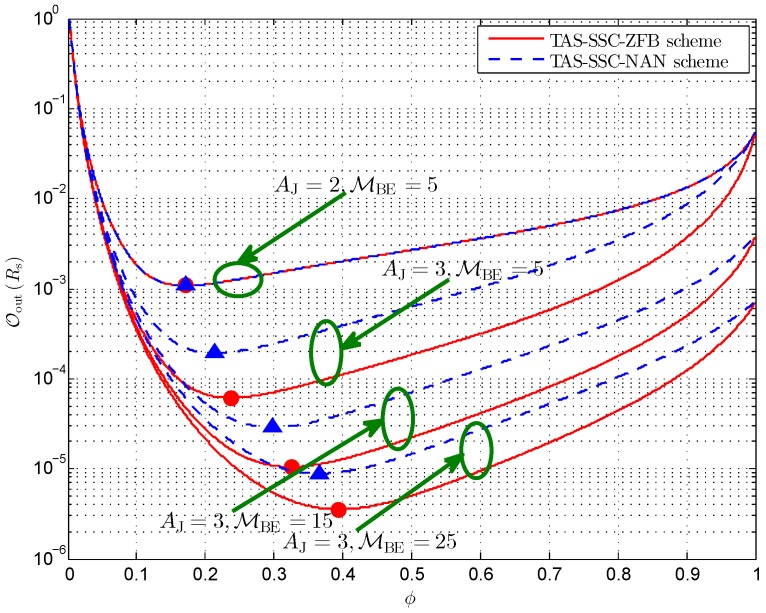
Secrecy outage probability versus different ϕ for $\gamma_{T}=10$ dB, $A_{A}=3,N_{B}=2,R_{s}=1$, and ${\bar{\gamma }}_{S}=20$ dB.

Figure 5 shows the secrecy outage probability versus different power allocation between the BS and the cooperative jammer. Considering a given switching threshold, we can see that an optimal power allocation factor $\phi^{*}$ is found for both schemes. Specifically, the optimal factor $\phi^{*}$ shifts to the right when the number of antennas at the cooperative jammer increases (e.g., $A_{J}=2,M_{\mathrm{BE}}=5$ and $A_{J}=3,M_{\mathrm{BE}}=5$). This reveals that less power is allocated at the cooperative jammer since this antenna configuration improves the capabilities of jamming. Once again, we can see that the TAS-SSC-ZFB scheme and the TAS-SSC-NAN scheme yield the same secrecy performance when the cooperative jammer is equipped with only two antennas. Furthermore, the optimal $\phi^{*}$ shifts to the left for both schemes when ${\bar{\gamma }}_{E}$ increases. This indicates that more power is necessary to be allocated for the jamming signal to achieve the minimum $O_{\mathrm{out}}\left(R_{s}\right)$ in view of higher ${\bar{\gamma }}_{E}$.

**Figure 6:**
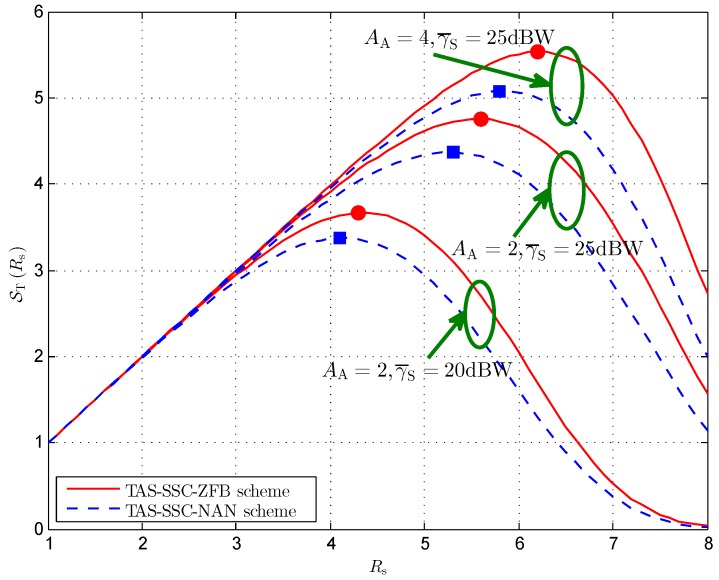
Effective secrecy throughput versus different $R_{s}$ for $A_{J}=3$, $\gamma_{T}=10$ dB, $N_{B}=2$, ${\bar{\gamma }}_{B}/{\bar{\gamma }}_{E}=10$, and ϕ=0.5.

Figure 6 plots the effective secrecy throughput versus different secrecy rates for a given switching threshold and power allocation factor. In this figure, it is found that $S_{T}$ first increases and then decreases as $R_{s}$ increases, which demonstrates that there exists an optimal $R_{s}^{*}$ point to achieve the largest effective secrecy throughput. To begin with, we concentrate on the impact of the number of transmit antenna elements $A_{A}$ and the total transmit power ${\bar{\gamma }}_{S}$. We readily observe that a larger effective secrecy throughput is obtained while either $A_{A}$ or ${\bar{\gamma }}_{S}$ increases. Moreover, it can be seen that the TAS-SSC-ZFB scheme slightly outperforms the TAS-SSC-NAN scheme in the medium to high regime of $R_{s}$. Furthermore, the optimal $R_{s}^{*}$ shifts to the right while considering that either $A_{A}$ or ${\bar{\gamma }}_{S}$ improves. For instance, considering the cases $A_{A}=2,{\bar{\gamma }}_{S}=20$ dBW and $A_{A}=2,{\bar{\gamma }}_{S}=25$ dBW, we see that increasing ${\bar{\gamma }}_{S}$ from 20 dBW to 25 dBW leads to enhancement of $R_{s}^{*}$ from 4.1 to 5.3 for the TAS-SSC-NAN scheme as well as from 4.3 to 5.6 for the TAS-SSC-ZFB scheme. Likewise, considering the cases $A_{A}=2,{\bar{\gamma }}_{S}=25$ dBW and $A_{A}=4,{\bar{\gamma }}_{S}=25$ dBW, we find that increasing $A_{A}$ from 2 to 4 increases $R_{s}^{*}$ from 5.3 to 5.8 for the TAS-SSC-NAN scheme as well as from 5.6 to 6.2 for the TAS-SSC-ZFB scheme. These observations indicate that the BS supports a larger secrecy rate for higher $A_{A}$ and ${\bar{\gamma }}_{S}$.

**Figure 7:**
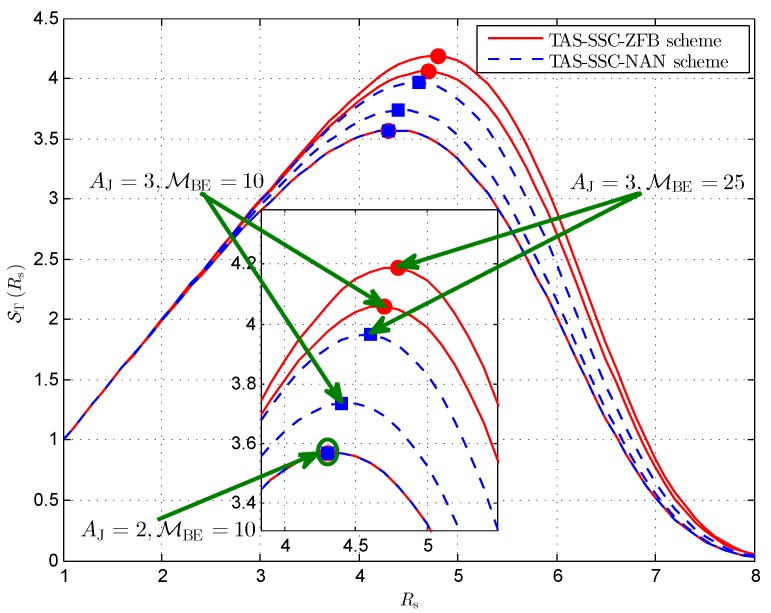
Effective secrecy throughput versus different $R_{s}$ for $A_{A}=3$, $\gamma_{T}=10$ dB, $N_{B}=2$, ${\bar{\gamma }}_{S}=20$ dBW, and ϕ=0.5.

We now focus on the impact of the number of antenna elements at the jammer $A_{J}$ or the average SNR of legitimate channel ${\bar{\gamma }}_{E}$. As can be obviously revealed from Figure 7, decreasing $A_{J}$ or increasing ${\bar{\gamma }}_{E}$ results in a reduction in the effective secrecy throughput. Moreover, we find that $R_{s}^{*}$ shifts to the left when $A_{J}$ decreases or ${\bar{\gamma }}_{E}$ increases. Considering the cases $A_{J}=2,M_{\mathrm{BE}}=10$ and $A_{J}=3,M_{\mathrm{BE}}=10$, it can be seen that reducing $A_{J}$ from 3 to 2 decreases $R_{s}^{*}$ from 4.4 to 4.3 for the TAS-SSC-NAN scheme and from 4.7 to 4.3 for the TAS-SSC-ZFB scheme. Similarly, comparing the cases of $A_{J}=3,M_{\mathrm{BE}}=10$ and the case of $A_{J}=3,M_{\mathrm{BE}}=25$, it can be found that increasing ${\bar{\gamma }}_{E}$ from ${\bar{\gamma }}_{B}/25$ to ${\bar{\gamma }}_{B}/10$ decreases $R_{s}^{*}$ from 4.6 to 4.4 for the TAS-SSC-NAN scheme and from 4.8 to 4.7 for the TAS-SSC-ZFB scheme. These observations indicate that the BS supports a lower secrecy rate for higher ${\bar{\gamma }}_{E}$ and smaller $A_{J}$.

## 5. Conclusions

In this paper, we designed the secure transmission in the SSC based WSNs with TAS and cooperative jamming. Specifically, the TAS scheme was adopted at the BS, and the ZFB scheme as well as the NAN scheme were, respectively, explored at the cooperative jammer to further improve the security of the considered network. In doing so, we derived the novel exact closed-form expressions for the secrecy outage probability and the effective secrecy throughput to evaluate the secrecy performance achieved by both schemes. Additionally, numerical results were presented to validate the analysis of the proposed schemes and provide insights into the impact of key system parameters on the secrecy performance. Finally, it was revealed that the TAS-SSC-ZFB scheme outperforms the TAS-SSC-NAN scheme in terms of the secrecy outage probability and the effective secrecy throughput, while the TAS-SSC-NAN scheme is more robust than the TAS-SSC-ZFB scheme.
